# Guidance of empirical antimicrobial therapy by surveillance cultures in high-risk neutropenic patients: a retrospective cohort study

**DOI:** 10.1186/s13756-022-01198-5

**Published:** 2022-12-18

**Authors:** Jara R. de la Court, Jarom Heijmans, Jennifer Huynh, Elske Sieswerda, Nick A. de Jonge, Karin van Dijk, Kim C. E. Sigaloff, Rogier P. Schade

**Affiliations:** 1 Division of Infectious Diseases, Department of Internal Medicine, Amsterdam UMC, Vrije Universiteit Amsterdam, University of Amsterdam, Amsterdam, The Netherlands; 2 Department of Medical Microbiology and Infection Prevention, Amsterdam UMC, Vrije Universiteit Amsterdam, University of Amsterdam, Amsterdam, The Netherlands; 3 Department of Haematology, Amsterdam UMC, Vrije Universiteit Amsterdam, University of Amsterdam, Amsterdam, The Netherlands; 4 Department of Medical Microbiology, University Medical Center Utrecht, Utrecht University, Utrecht, The Netherlands; 5 Julius Center for Health Sciences and Primary Care, University Medical Center Utrecht, Utrecht University, Utrecht, The Netherlands

## Abstract

**Background:**

In neutropenic patients, bloodstream infections (BSI) significantly contribute to morbidity and mortality. Appropriate empirical antibiotic therapy (EAT) of BSI is essential, at the same time overconsumption of very broad-spectrum antibiotics should be avoided. We investigated: (1) whether surveillance cultures can predict BSI with third-generation cephalosporin –resistant Enterobacterales and *Pseudomonas aeruginosa* (3GC-R), (2) the effect of inappropriate empirical antimicrobial therapy (IEAT) on clinical outcome and (3) the potential reduction of carbapenem use when using surveillance cultures to guide therapy.

**Methods:**

Retrospective study of adult patients with haematological malignancies with febrile episodes during chemotherapy-induced high-risk neutropenia in whom surveillance cultures were collected weekly. IEAT was defined as the absence of in vitro susceptibility of blood-isolates to the administered EAT. Clinical outcome (ICU admission and death) was evaluated within 30 days.

**Results:**

A total of 673 febrile episodes occurred among 372 high-risk neutropenic patients. BSI was present in 20.1% (135/673), of which 25.9% (35/135) were due to Enterobacterales and *P. aeruginosa.* Of these, 17/35 were 3GC-R and 70.6% (12/17) were preceded by 3GC-R colonization. Negative predictive value of surveillance cultures for 3GC-R BSI was 99.1%. IEAT due to (3GC-R) BSI was not significantly associated with clinical outcome. Using surveillance cultures to guide EAT could potentially reduce carbapenem use by 82.8%, when compared to standard EAT with carbapenem.

**Conclusions:**

This retrospective analysis shows that in patients with high-risk neutropenia, surveillance cultures can potentially reduce the use of carbapenems with infrequent IEAT for 3GC-R BSI and no negative impact on clinical outcome.

**Supplementary Information:**

The online version contains supplementary material available at 10.1186/s13756-022-01198-5.

## Introduction

Infections with Gram-negative bacteria (GNB), especially Enterobacterales and *Pseudomonas aeruginosa* (EP), are amongst the most feared complications in chemotherapy-induced neutropenic patients. Prompt initiation of empirical antibiotic therapy (EAT) with broad-spectrum antibiotics reduces morbidity and mortality for patients with febrile neutropenia [1]. At the same time, this population is at risk for overconsumption of very broad-spectrum antibiotics, with the associated threat of increasing bacterial resistance prevalence and other adverse events [2, 3].

The current European guidelines for empirical antibacterial therapy for febrile neutropenic patients (ECIL4) recommend antipseudomonal β-lactams, i.e. ceftazidime, piperacillin-tazobactam, cefepime and carbapenems [4], and advise to take local resistance epidemiology and patients’ risk factors for colonization with resistant bacteria into account when selecting an empiric regimen [5]. Just now, a Dutch guideline for the treatment of febrile neutropenic patients was published that clearly recommends carbapenems as second-choice EAT [6]. However, before the publication of this guideline, there was no consensus on the restrictive use of carbapenems in the Dutch febrile neutropenic patient population and carbapenems were used as first-choice EAT in 3/8 academic haematology centres [7]. In many hematological centers, colonization status is systematically evaluated in high-risk neutropenic patients by weekly performance of surveillance cultures [6–8]. These routine surveillance cultures can potentially be used to withhold very broad-spectrum antibiotic therapy (e.g. carbapenems) from patients without colonization of third-generation cephalosporin resistant Enterobacterales and *P.aeruginosa* (3GC-R). Current data on this assumption is, however, inconsistent [8, 9]. The aim of this study was to investigate the predictive value (sensitivity, specificity, positive predictive and negative predictive value) of surveillance cultures and to assess the risk of inappropriate empirical antimicrobial therapy (IEAT) and the effect of IEAT on clinical outcome (Intensive Care Unit (ICU) admission and death) in the two different treatment centres studied (i.e. standard meropenem-treating centre and standard ceftazidim-treating centre). Furthermore, we examined the potential risk and benefits when using surveillance cultures to guide the selection of EAT (e.g. ceftazidime versus carbapenems) by calculating the potential risk of IEAT and the potential carbapenem reduction if such an surveillance culture guided EAT approach was used in this study population.

## Methods

### Study design and setting

This retrospective cohort study was conducted in two university medical centres that had different EAT regimes for febrile episodes in high-risk neutropenic patients, in Amsterdam, the Netherlands,: the VU University Medical Centre, a 730-bed tertiary care centre (referred to as meropenem-treating centre (MTC)), and the Academic Medical Centre a 1002-bed tertiary care centre (referred to as ceftazidime-treating centre (CTC)). The different antibiotic policies in the centres are described in Additional file 1: Table S1.

### Patients and data collection

We screened all neutropenic patients > 18 year, who received antibiotic prophylaxis, admitted to the haematology wards during the following period: march 2016 to april 2019 in MTC and October 2015 to december 2019 in CTC. All patients who received chemotherapy for a haematological malignancy resulting in protracted neutropenia (< 500 neutrophils/μL for > 7 days), further referred to as high-risk neutropenic patients, with at least one febrile episode were included. We excluded all patient who were transferred to another hospital during treatment. We also excluded all patients that opted out of chemotherapy before neutropenia occurred and all patients in which fever did not occur. From the included patients we gathered the following variables: age, gender, underlying hematologic malignancy, type and start date of the chemotherapeutic course, neutrophil count, white blood cell count and differential, presence of peripheral of central venous catheter, total parenteral nutrition, ICU transfer and mortality.

### Definitions

**Fig. 1:**
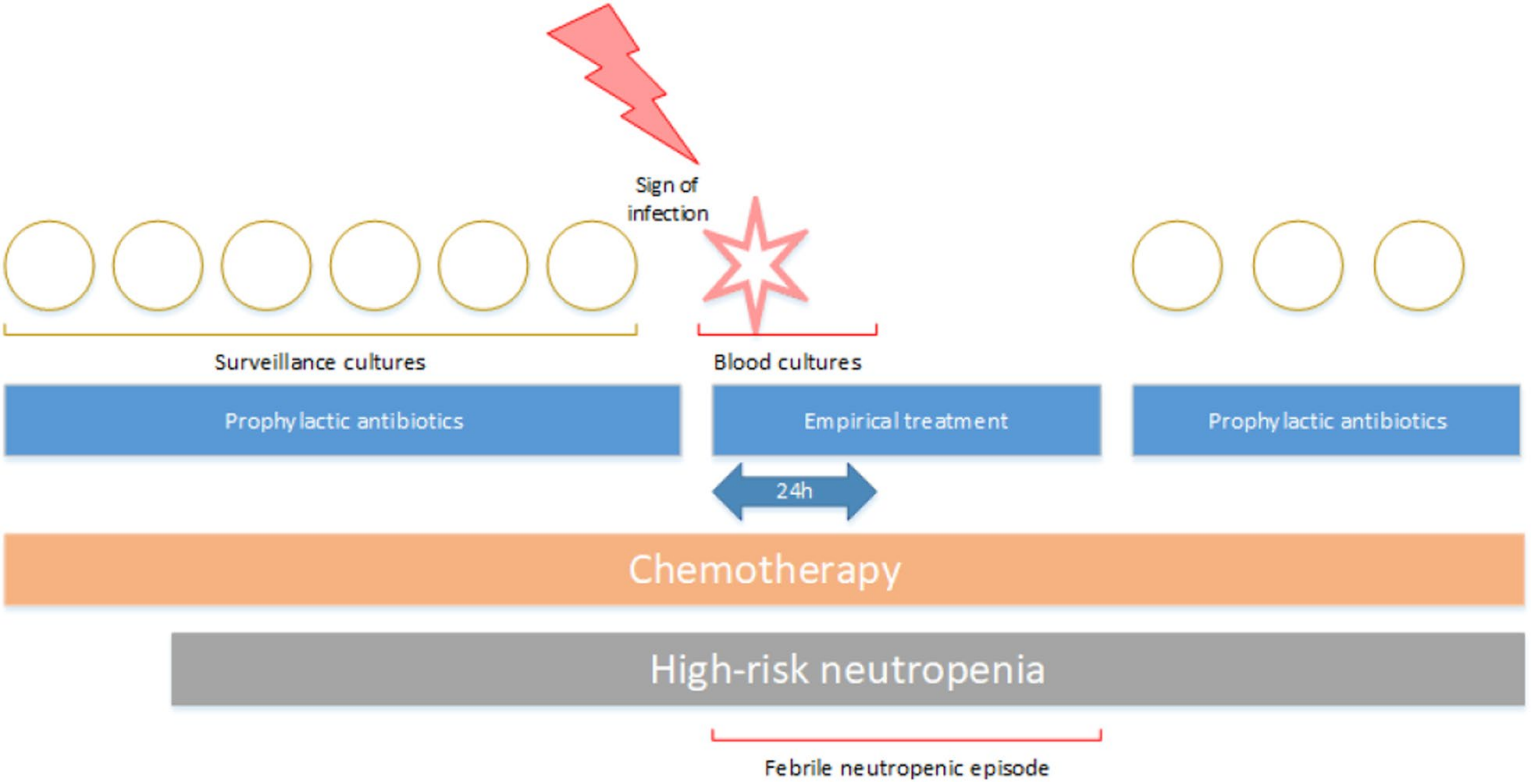
Schematic overview FN episode.

A febrile episode was defined as an episode during high-risk neutropenia, in which diagnostic blood cultures were drawn due to clinical signs and symptoms of infection (fever, hypotension and/or chills) and in which empirical antibiotic therapy was initiated within 48 h (Fig. 1). Fever was defined as a temperature of ≥ 38.3 °C measured once, or ≥ 38.0 °C measured multiple times during one hour.

Subsequent febrile episodes could be included when the systemic antibiotics had been halted and prophylactic antibiotics were restarted for at least 24 h. In case of clinical signs and symptoms of infection (fever, hypotension and/or chills) at least two blood cultures were drawn according to local protocols in both centres. All blood cultures drawn at onset of fever were included in the analysis. Blood cultures drawn after ≥ 24 h of empirical treatment were excluded from the analysis to ensure selection of the EAT, as treatment is often optimized thereafter based on culture results or clinical course of the infection. We included BSI with coagulase-negative staphylococci (CNS) according to the CDC criteria for laboratory confirmed bloodstream infection with common commensals, which means that CNS should be identified by a culture from two or more blood specimens collected on separate occasions [11]. IEAT was defined as EAT that did not include at least one in vitro active antibiotic against the isolated microorganism in the BSI. In case of infections with vancomycin susceptible CNS and enterococci species, administration of vancomycin within 48 h after onset of fever was regarded as adequate treatment (as per protocol in both centres). The duration of neutropenia was calculated by counting days from the last day before neutrophil levels dropped < 500/μL until neutrophil recovery (≥ 500 neutrophils/μL). When patients died during neutropenia the end of the neutropenic episode was the date of death. In the absence of an absolute neutrophil count, the white blood cell and differential were used to define neutropenic period.

### Microbiological procedures

Of the included patients, all surveillance and blood-isolates gathered during the study period were extracted. In high-risk neutropenic patients from both centres, pharyngeal and rectal samples were obtained weekly, beginning one week before the start of chemotherapy until neutrophil recovery (> 500 neutrophils). In pharyngeal and rectal samples growth and susceptibility of *Pseudomonas aeruginosa* and aerobic gram-negative bacilli were reported. In rectal swabs drawn in CTC, growth and susceptibility of gram-negative bacilli were only reported in pathogens resistant to either cotrimoxazole, ciprofloxacin or both. In MTC, growth and susceptibility of *Candida* spp. were also reported, whereas in the CTC growth and names of yeast and susceptibility testing was only performed when requested by the treating physician or the clinical microbiologist.

For microorganisms without EUCAST breakpoints, or isolates without MIC values, the S/I/R interpretation as reported by the laboratory was used, if available. In this study 3GC-R was defined as: *P. aeruginosa* and/or Enterobacterales group I non-susceptible to ceftazidime according to EUCAST breakpoints or I + R interpretation as reported [12]. Enterobacterales group II (*Enterobacter cloacae complex*, *Klebsiella aerogenes* (formerly *E. aerogenes*), *Citrobacter freundii complex, Hafebrileia alvei, Serratia* spp., *Providencia* spp., *Morganella morganii*) were considered 3GC-R independent of MIC values due to inducible chromosomal AmpC β-lactamases [13, 14]. Extended-spectrum beta-lactamase (ESBL) production was confirmed by combination disc diffusion test with both cefotaxime and ceftazidime, with and without clavulanic acid (Rosco, Taastrup, Denmark), interpreted according to the Dutch national guideline[13]. Gram negative bacteria (GNB) were categorized as ‘carbapenem-resistant’ if reported intermediately susceptible or resistant (I + R) to meropenem or imipenem, and otherwise as ´non-carbapenem-resistant´ (i.e. carbapenem-susceptible or not reported, according to local protocols).

3GC-R colonization was defined as the presence of a 3GC-R in rectal or pharyngeal swabs obtained for surveillance purposes. Febrile episodes within 3–365 days after the retrieval of a 3GC-R in surveillance cultures were regarded as febrile episodes preceded by 3GC-R colonization. The time interval of 365 days was based upon ESBL-carriage in travellers in whom most (88.7%) decolonized within one year [15]. This relationship was investigated independent of the micro-organism involved. In other words, a 3GC-R was considered predictive of any 3GC-R microorganism, i.e. *E. coli* could predict a 3GC-R *K. pneumoniae*. When multiple isolates were available, the shortest interval between 3GC-R positive surveillance cultures and 3GC-R BSI was used to calculate the median time between (the latest known) colonization and BSI.

### Data-analysis and statistics

Continuous variables were presented as mean ± standard deviation if normally distributed, or as the median and interquartile range if skewed. Categorical variables were presented as proportions and/or counts. Sensitivity, specificity, positive (PPV) and negative predictive values (NPV) were calculated using standard formulas [16]. We performed three additional sensitivity analysis. In the first, we only included surveillance cultures positive for 3GC-R with co-resistance to either cotrimoxazole or ciprofloxacine, resembling the culture methods in CTC and identifying resistance against the standard prophylactic regimens. In the second, we only included ESBL-producing Enterobacterales (ESBL-E) to confirm that the relationship between colonization and BSI was independent of our definition of 3GC-R. In the third, we shortened the time interval between surveillance culture and febrile episode to one month. We included multiple dependent febrile episode per patients, therefore for the analyses of data we used Generalized Estimating Equations (GEE) with an independent correlation structure. In our GEE analyses we estimated the correlation between BSI, 3GC-R-EP BSI and IEAT and the dichotomous clinical outcome: ICU transfer/all-cause mortality within 30 days after onset of fever. We investigated confounding by age, sex and treatment centre. The potential reduction of carbapenem use was calculated as the proportion of febrile episodes not preceded by 3GC-R colonization therefore not necessitating empirical treatment with carbapenems in a surveillance culture guided EAT approach, compared to standard EAT with carbapenem. Data analysis was performed using R Statistical Software (version 4.0.3; R Foundation for Statistical Computing, Vienna, Austria).

## Results

### Patients

**Fig. 2:**
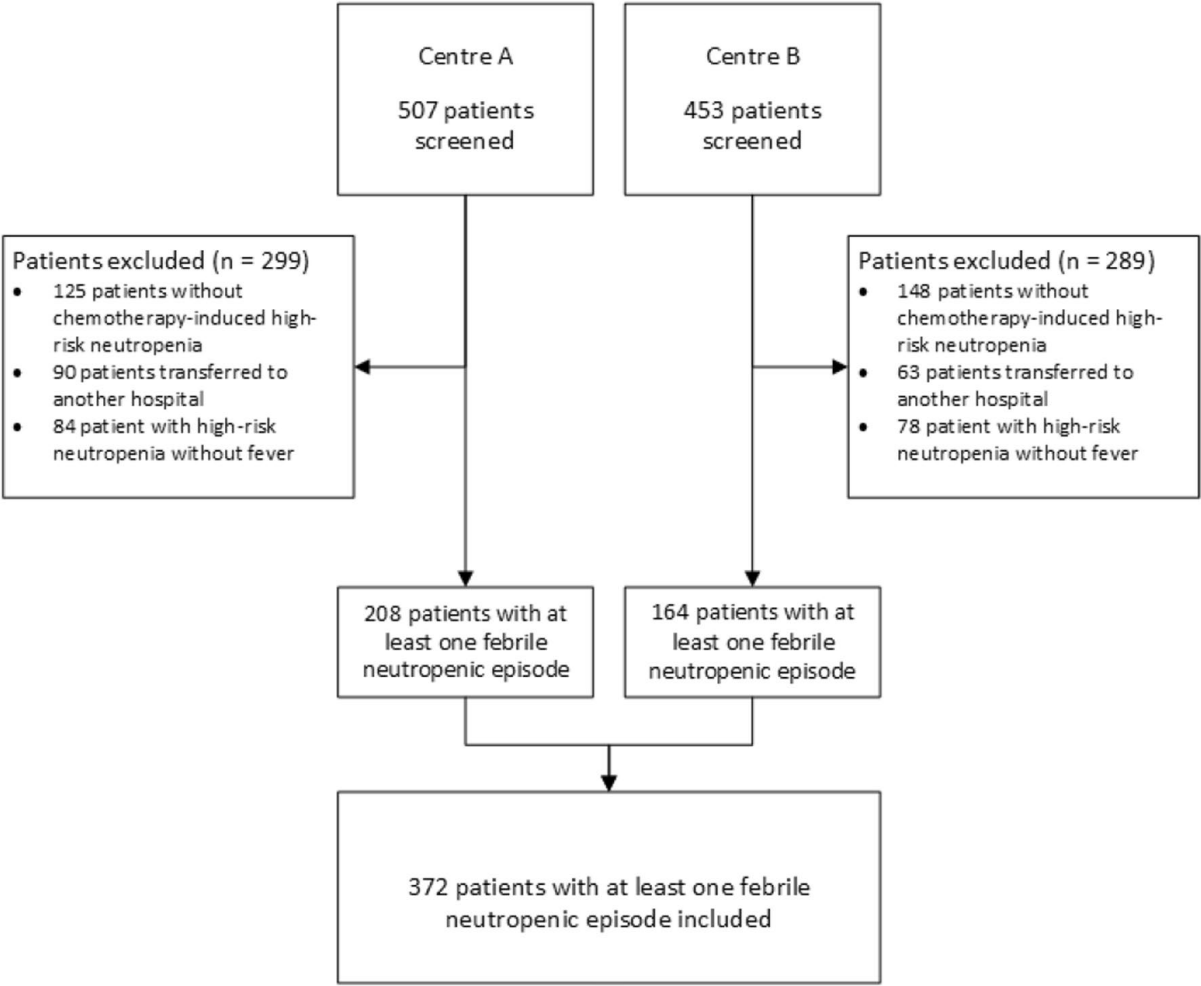
Flowchart

We screened 960 patients and included 372 high-risk neutropenic patients, with at least one febrile episode (MTC n = 208, CTC = 164, Fig. 2). Transfer to another hospital, after stem cell transplantation, was frequent in patients with lymphoma or multiple myeloma, whilst patients with leukaemia were mostly treated in the tertiary care departments studied. None of the included patients were lost to follow-up.

Patient characteristics (Table 1) showed a higher proportion of leukemic patients (67.8% versus 47.6%) and a longer duration of neutropenia (26 days versus 17 days) in MTC compared to CTC.

**Table 1:** Patient characteristics

	MTC	CTC	Total
	(N = 208)	(N = 164)	(N = 372)
Age (at inclusion)			
Median (IQR)	61.0 (16)	59.0 (15)	59.0 (16)
Sex			
Male, n (%)	121 (58.2)	104 (63.4)	225 (60.5)
Underlying haematologic malignancy, n (%)			
Acute leukaemia/blastic transformation	141 (67.8)	78 (47.6)	219 (58.7)
Acute lympoblastic leukemia	9 (4.3)	4 (2.4)	13 (3.5)
Chronic lymphoblastic leukemia	0 (0.0)	1 (0.6)	1 (0.3)
Lymphoma	24 (11.5)	34 (20.7)	58 (15.6)
Myelofibrosis	9 (4.3)	2 (1.2)	11 (3.0)
Plasma cell disorders	25 (12.0)	45 (27.4)	70 (18.8)
Received conditioning chemotherapy for stem cell transplantation	62 (29.8)	20 (12.2)	82 (22.0)
Amount of chemotherapy courses causing high-risk neutropenia included per patient			
1	108	112	220
2	50	26	76
3	44	21	65
4	4	5	9
5	2	0	2
Febrile neutropenic episodes	348	325	673
Febrile neutropenic episodes included per patient			
1	117	89	206
2	56	32	88
3	25	19	44
4	7	12	19
5	2	7	9
6	1	4	5
7	0	1	1
Central catheter, n (%)			
PICC	192 (92.3)	3 (1.8)	195 (52.4)
CVC	7 (3.3)	158(96.3)	165(44.4)
PICC and CVC	6 (2.9)	1 (0.6)	7 (1.9)
Unknown/Missing data	3 (1.4)	-	3 (0.8)
None	0 (0.0)	2 (1.2)	2 (0.5)
Total parenteral nutrition, n (%)	61 (29.3)	104 (63.4)	165 (44.4)
Neutropenic episode duration in days, median (IQR)	26 (14)	17 (14)	23 (16)
ICU transfer/all-cause mortality within 30 days	35 (16.8)	34 (20.7)	69 (18.5)

### Blood stream infection

During the study period 372 patients had 673 febrile episodes (348 MTC and 325 CTC). Blood cultures drawn within 48 h (and with < 24 h of empirical therapy) after the onset of fever were positive in 20.1% (135/673) of the febrile episodes (Table 2). Of these BSI 25.9% (35/135) were due to Enterobacterales and *P. aeruginosa*. EAT for these febrile episodes is described in Additional file 1: Table S3. IEAT was present in 4.0% (27/673) of febrile episodes (Table 2). In the MTC the majority of IEAT was associated with candidemias (11/21), whereas the majority (4/6) of IEAT in the CTC was due to gram-negative bacteria.

**Table 2:** Distribution of pathogens and IEAT in initial BSI

Pathogens	MTC n (%)	IEAT	CTC n (%)	IEAT	Total	Total IEAT
*Candida* spp.	8 (9.5)	7	0 (0.0)	0	8 (5.9)	7
Gram-positive bacteria	32 (38.1)	5	27 (52.9)	1	59 (43.7)	6
*Coagulase negative staphylococci*	21	5	11	1	32	6
*Enterococcus* spp.	9	0	6	0	15	0
*Streptococcus* spp.	1	0	10	0	11	0
Other gram-positive bacteria	1	0	0	0	1	0
Gram-negative bacteria	34 (40.5)	4	14 (27.5)	4	48 (35.6)	8
Enterobacterales not constitutively producing AmpC						
*Escherichia coli*	12	0	9	0	21	0
*Klebsiella pneumoniae*	4	0	0	0	4	0
*Proteus mirabilis*	1	0	1	0	2	0
Enterobacterales constitutively producing AmpC						
*Citrobacter freundii*	0	0	1	1	1	1
*Enterobacter cloacae/asburiae*	2	0	0	0	2	0
*Serratia marcescens*	3	0	0	0	3	0
*Pseudomonas aeruginosa*	2	1	0	0	2	1
*Acinetobacter* spp.	2	0	2	2	4	2
*Stenotrophomonas maltophilia*	2	1	0	0	2	1
Other non-fermentative gram-negative bacteria						
*Chryseobacterium indologens*	1	0	0	0	1	0
*Pseudomonas* spp.	2	1	0	0	2	1
*Rhizobium radiobacter*	1	0	0	0	1	0
*Sphingomonas paucimobilis*	2	1	0	0	2	1
Other gram-negative bacteria						
*Capnocytophaga sputigena*	0	0	2	1	2	1
Polymicrobial	10 (11.9)	5**	10 (19.6)	1***	20 (14.8)	6
Total	84 (100)	21	51 (100)	6	135 (100)	27

**4/5 were positive with *Candida* spp. combined with bacterial pathogens; ***BSI with both *Achromobacter xylosoxida* and *P. aeruginosa* in one blood culture

Besides the bacteria described in Table 2, other GNB blood-isolates (non Enterobacterales or *P.aeruginosa)* also resistant to ceftazidime were excluded from the analysis. All GNB blood-isolates and their drug resistance patterns are listed in Additional file 1: Table S2.

### 3GC-R colonization and predictive value of 3GC-R colonization

3GC-R colonization was identified in 33.2% (69/208) and 11.0% (18/164) patients, in MTC and CTC respectively. In the analysis, in which only 3GC-R isolates with co-resistance to either cotrimoxazole or ciprofloxacine were included, 13.4% (50/372) patients were colonized with 3GC-R with co-resistance to either cotrimoxazole or ciprofloxacine.

In MTC, 69 patients with 3GC-R colonization developed 91 febrile episodes. In CTC, 18 patients with 3GC-R colonization developed 25 febrile episodes (Table 3). In total, 17.2% (116/673) of febrile episodes were preceded by 3GC-R colonization and in 10.3% (12/116) of these 3GC-R BSI was identified. Thereby reducing the the risk of IEAT from 2.5% (17/673) to 0.7% (5/673) when relying on surveillance cultures (Table 4). During febrile episodes in patients without 3GC-R colonization, 3GC-R BSI was present in 0.9% (5/557). Overall, unanticipated 3GC-R BSI occurred in 0.7% (5/673) of febrile episodes.

**Table 3:** Predictive value of 3GC-R in surveillance cultures, FN episodes of patients with or without 3GC-R colonization and/or with 3GC-R BSI

	3GC-R BSI	No 3GC-R BSI	Total
3GC-R colonization	12	104	116
No 3GC-R colonization	5	552	557
Total	17	656	673

Accuracy: 0.84, 95% CI: 0.81–0.87

Sensitivity: 70.6%

Specificity: 84.1%

PPV*: 10.3%

NPV*: 99.1%

*These values are dependent on disease prevalence

**Table 4:** Predictive value of ESBL-E in surveillance cultures, FN episodes of patients with or without ESBL-E colonization and/or with ESBLE-E BSI

	ESBL-E BSI	No ESBL-E BSI	Total
ESBL-E colonization	6	39	45
No ESBL-E colonization	2	626	628
Total	8	665	673

Accuracy: 0.94, 95% CI: 0.91 – 0.96

Sensitivity: 75.0%

Specificity: 94.1%

PPV*: 13.3%

NPV*: 99.7%

*These values are dependent on disease prevalence

Sensitivity and negative predictive value of 3GC-R in surveillance cultures for BSI with 3GC-R were 70.6% and 99.1% respectively. Median time from the latest known surveillance culture positive with 3GC-R to the episode of 3GC-R BSI was 5 days (IQR = 3). In 2/17 BSI no phenotypically matching pathogen had been found in prior surveillance cultures: In one patient with a *K.pneumoniae* BSI, only a *C.freundii* was demonstrated in the prior surveillance cultures, and in the other patient, prior to an *E.cloacae* BSI, an ESBL-producing *E.coli* was demonstrated in the surveillance cultures. When only including surveillance cultures one month prior to the febrile episode, the sensitivity would decrease to 64.7%. This decrease was due to one 3GC-R BSI, preceded by a 3GC-R surveillance culture just over a month (39 days) prior to the febrile episode.

We also provide a contingency table for ESBL-producing Enterobacterales, which resulted in slightly higher sensitivity and negative predictive value compared to 3GC-R, i.e. sensitivity of 75% compared to 70.6% and a NPV 99.7% compared to 99.1% (Table 4).

### Mortality/ICU transfer

In 18.5% (69/372) high-risk neutropenic patients, the composite outcome of all-cause mortality/ICU transfer within 30-days after fever occurred. The composite outcome occurred in 14.4% (97/673) of febrile episodes (Additional file 1: Table S4). Additional file 1: Table S4 shows the OR and P-values derived from the GEE analysis. None of the covariates (BSI, 3GC-R-EP BSI and IEAT) significantly influenced the risk of all-cause mortality/ICU transfer, correction for age, sex and treatment centre did not alter these results. Although IEAT was present in 4.0% (27/673) febrile episodes, the majority 81.5% (22/27) of BSI with IEAT received appropriate antimicrobial treatment within 72 h of BSI onset.

### Potential reduction of carbapenem use

With a surveillance culture guided empirical therapy approach, in 17.2% (116/673) of febrile episodes—preceded by 3GC-R colonization—empirical therapy with carbapenems would have been given. This would lead to a reduction of 82.8% of carbapenem use compared to standard empirical treatment with carbapenems.

## Discussion

In this two-centre retrospective study of 372 patients with 673 high-risk chemotherapy induced febrile neutropenic episodes, the sensitivity and NPV of surveillance-cultures for 3GC-R BSI were 70.6% and 99.1%, respectively. IEAT occurred in 4% of febrile episodes and was due to 3GC-R BSI in one patient in the ceftazidime-treating centre. IEAT was not significantly associated with the composite clinical outcome measure ICU transfer/all-cause mortality within 30 days after onset of fever.

We hypothesized that a surveillance culture guided EAT approach could be used to select appropriate empirical antimicrobial therapy in a high-risk neutropenic patient population, based on prior colonization status. In line with this hypothesis, we found a high NPV of surveillance cultures: in patients without 3GC-R colonization, 99.1% did not have BSI with 3GC-R. In our study, unanticipated BSI with 3GC-R occurred in 0.7% (5/673) of febrile episodes. The low occurrence of unanticipated 3GC-R bacteraemia is in line with previous studies [17–25]. In these studies, the sensitivity of colonization with Multi-Drug-Resistant (MDR) bacteria for MDR-BSI in the hematologic patient population ranged from 45 to 91%. High negative predictive values of ESBL-E colonization for ESBL-E bacteraemia have been described (73.9–99.8%), indicating that bacteraemia in the absence of prior colonization is uncommon [17, 19, 20, 22, 23].

In the ceftazidime-treating centre one unanticipated 3GC-R BSI resulted in IEAT whereas none of the 3GC-R BSI resulted in IEAT in the meropenem-treating centre, because these GNB were all carbapenem-susceptible. However, when all pathogens were taken into account, IEAT occurred more often in the meropenem-treating centre compared to the ceftazidime-treating centre: 25.0% (21/84) versus 11.8% (6/51). This was mostly due to candidemias: 11/21 IEAT in the meropenem-treating centre were candidemias. There were several differences between the centres that could explain the different prevalence of candidemia; the meropenem-treating centre had a higher prevalence of patients with acute leukemia/blastic transformation, a longer median duration of neutropenia and a higher exposure to carbapenems compared to the ceftazidime-treating centre [25].

In our study, IEAT was not significantly associated with ICU transfer/all-cause mortality within 30 days after onset of fever. Likewise, in a study by Martinez-Nadal et al. IEAT did not result in a significant increase in mortality, with the exception of *P. aeruginosa* BSI [26]. Because IEAT only occurred in 4% of the febrile episodes studied it is possible we were not able to detect a clinical relevant effect on ICU transfer/mortality within 30 days. Another explanation could be the short time-to-positivity of blood cultures in this patient population [27]. As the vast majority of blood cultures are positive within 24 h, it is not surprising that in our study the majority 81.5% (22/27) of BSI with IEAT received appropriate antimicrobial treatment within 72 h of BSI onset. From the study by Tang et al. we know that patients who did not receive appropriate antibiotics within 72 h of BSI onset did have a significantly increased 7-day mortality [28]. Therefore the duration until appropriate treatment was given could have influenced the association between IEAT and clinical outcome.

Compared to standard empirical carbapenems, a surveillance culture guided EAT approach, in which standard treatment with ceftazidime (or another antipseudomonal beta-lactam) is given with the exception of patients colonized with 3GC-R, can reduce carbapenem use. If our total study population would receive empirical carbapenem in case of fever, the number needed to treat with carbapenem to appropriately treat one 3GC-R BSI would be 39.6 (673/17). Using a surveillance guided EAT approach, 17.2% (116/673) of febrile episodes would have been treated with carbapenems based on the results of surveillance cultures. Surveillance culture guided EAT reduces the risk of IEAT due to 3GC-R BSI from 2.5%, (17/673) to 0.7% (5/673). In other words, with this approach carbapenem usage can potentially be reduced by 82.8% compared to a standard empirical carbapenem strategy, with the risk that 0.7% of febrile episodes receive IEAT due to unanticipated 3GC-R BSI.

The strengths of this study are the comprehensive clinical and microbiological dataset in which we included all febrile episodes of the study patients. Furthermore, the two-centre approach allowed a larger sample size for more generalizable findings. Despite the inclusion of many febrile episodes, the occurrence of 3GC-R BSI was infrequent, limiting the power of a subgroup analysis of these BSIs. We used a strict definition of 3GC-R BSI excluding other, mostly non-fermentative, GNB. This approach allowed us to identify patients colonized with pathogens that would benefit from an escalation to carbapenems whilst excluding pathogens that are neither ceftazidime nor carbapenem susceptible (e.g. *Stenotrophomonas maltophilia*). Furthermore, this approach excluded GNB BSI with low-virulence and weak direct attributable mortality [29]. Another limitation was the microbiological method used in the ceftazidime-treating centre for detecting pathogens in surveillance cultures. By only including pathogens with co-resistance to either cotrimoxazole or ciprofloxacine we could have underestimated 3GC-R or ESBL-E colonization. However, even with possibly under detected 3GC-R colonization the negative predictive value of surveillance cultures was high and only one patient received IEAT due to 3GC-R. Lastly, this study was performed in two centres from the same geographical region with a low background prevalence of resistance. Therefore, the global epidemiology and prevalence of multidrug resistance is likely to vary in centres from other regions. In countries with a higher prevalence of 3GC-R GNB the benefits of a surveillance culture guided empirical therapy approach could be less evident. As the prevalence of 3GC-R GNB colonization increases the potential reduction of carbapenem use with this approach reduces. Furthermore, with an increase in 3GC-R GNB BSI the risk of IEAT due to unanticipated 3GC-R BSI increases. This current study shows the potential benefit of a surveillance culture guided therapy approach in a setting with a relatively low background prevalence of resistance. In any case, this study offers a concept for the way in which surveillance cultures can be used to guide antibiotic policy. This concept should, however, be reconsidered in one's own epidemiological setting. Furthermore, one could apply this concept while using other agents as EAT such as cefepime or piperacillin/tazobactam. As it is likely that the predictive value of surveillance cultures does not depend on the specific agent used as EAT. Besides, the potential reduction of carbapenem use does not take into account other reasons for carbapenem use, such as antibiotic allergies, severely ill patients transmitted to the ICU with a de-escalation policy or when a neutropenic enterocolitis is suspected (e.g. abdominal pain combined with severe colitis on CT scans).Prospective evaluation of the safety and benefits of a surveillance culture guided empirical therapy approach are therefore warranted.

## Conclusion

In this retrospective analysis we found that among patients with high-risk neutropenia, surveillance cultures had a negative predictive value of 99.1%, indicating that in patients without prior colonization 3GC-R BSI is rare. Furthermore, IEAT did not worsen clinical outcomes. In a surveillance culture guided empirical therapy approach carbapenem use can be reserved for febrile episodes preceded by 3GC-R colonization, reducing the use of carbapenem in this population by 82.8%.

## Supplementary Information

**Additional file 1: Table S1.** Antimicrobial policies in both centres. **Table S2.** Gram-negative organisms in initial blood cultures, including GNB from polymicrobial BSI; distribution and resistance patterns. **Table S3.** Empirical antibiotic therapy. Table S4. Generalized Estimating Equations analysis of BSI, 3GC-R EP BSI and IEAT for the composite outcome all-cause mortality/ ICU-transfer within 30-days after FN onset.

## Data Availability

We will share data, which have been used for this publication, upon reasonable request.
