# Supplementary material for: Guidance of empirical antimicrobial therapy by surveillance cultures in high-risk neutropenic patients: a retrospective cohort study
Source: Antimicrob Resist Infect Control. 2022 Dec 18;11:160. doi: 10.1186/s13756-022-01198-5 (PMC9759862; doi:10.1186/s13756-022-01198-5)
Supplement: Supplementary file 1 — Additional file 1: Table S1. Antimicrobial policies in both centres. Table S2. Gram-negative organisms in initial blood cultures, including GNB from polymicrobial BSI; distribution and resistance patterns. Table S3. Empirical antibiotic therapy. Table S4. Generalized Estimating Equations analysis of BSI, 3GC-R EP BSI and IEAT for the composite outcome all-cause mortality/ ICU-transfer within 30-days after FN onset. [file 13756_2022_1198_MOESM1_ESM.docx]

Supplementary table 1; Antimicrobial policies in both centres

|  | **Meropenem-treating centre** | **Ceftazidime-treating centre** |
| --- | --- | --- |
| Empirical treatment | meropenem 1000 mg t.i.d.  (continued for 9 days in case of persistent fever of unknown origin) | ceftazidime 2000 mg t.i.d.  (continued for 2 days in case of persistent fever of unknown origin) |
| Antibacterial prophylaxis | ciprofloxacin 500 mg b.i.d. +tobramycin 120 mg t.i.d. | ciprofloxacin 500 mg b.i.d. |
| Antifungal prophylaxis | fluconazole 50 mg q.d. | amphotericin B suspension 500 mg q.i.d |
| In case of mucositis addition of | feneticillin 250 mg q.i.d./ amoxicillin 500 mg t.i.d. | feneticillin 250 mg q.i.d./ amoxicillin 500 mg t.i.d. |
| In case of/suspicion of central-venous-line infection; addition of | vancomycin 1000 mg b.i.d. (adjusted with therapeutic drug monitoring | amoxicillin/clavulanic acid 500/125 mg t.i.d. or vancomycin 1000 mg b.i.d. (adjusted with therapeutic drug monitoring |

Supplementary table 2; Gram-negative organisms in initial blood cultures, including GNB from polymicrobial BSI; distribution and resistance patterns.

|  |  | Resistance to antibiotic/antibiotic group  N = resistant isolates/total number of isolates of that organism with available susceptibility test | | | |
| --- | --- | --- | --- | --- | --- |
| Meropenem-treating centre | **Gram-negative bacteria** | **Quinolones** | **Co-trimoxazole** | **Ceftazidime** | **Carbapenems** |
|  | **Enterobacterales not constitutively producing AmpC** |  |  |  |  |
|  | *Escherichia coli* | 12/12 | 8/12 | 3/12 | 0/12 |
|  | *Klebsiella pneumoniae* | 3/4 | 3/4 | 3/4 | 0/4 |
|  | *Proteus mirabilis* | 0/1 | 1/1 | 0/1 | 0/1 |
|  | **Enterobacterales constitutively producing AmpC*** |  |  |  |  |
|  | *Enterobacter cloacae/asburiae* | 0/2 | 0/2 | 0/2 | 0/2 |
|  | *Serratia marcescens* | 0/4 | 0/4 | 0/4 | 0/4 |
|  | ***Pseudomonas aeruginosa*** | 2/3 | NA | 2/3 | 2/3 |
|  | ***Acinetobacter* spp.** | 0/2 | 0/2 | 0/1 | 0/2 |
|  | ***Stenotrophomonas maltophilia*** | NA | 1/2 | NA | NA |
|  | **Other non-fermentative gram-negative bacteria** |  |  |  |  |
|  | *Chryseobacterium indologenes* | 1/1 | 0/1 | 1/1 | 1/1 |
|  | *Pseudomonas* spp. | 1/2 | 1/1 | 0/1 | 1/2 |
|  | *Rhizobium radiobacter* | 0/1 | 0/1 | 1/1 | 0/1 |
|  | *Sphingomonas paucimobilis* | 0/2 | 0/2 | 1/1 | 1/2 |
|  | **Total** | **19/34** | **13/34** | **11/34** | **5/34** |
| Ceftazidime-treating centre | **Enterobacterales not constitutively producing AmpC** |  |  |  |  |
|  | *Escherichia coli* | 12/12 | 10/12 | 2/12 | 0/12 |
|  | *Proteus mirabilis* | 0/1 | 1/1 | 0/1 | 0/1 |
|  | **Enterobacterales constitutively producing AmpC*** |  |  |  |  |
|  | *Citrobacter freundii* | 1/1 | 1/1 | 0/1 | 0/1 |
|  | ***Pseudomonas aeruginosa*** | 0/1 | NA | 0/1 | NA |
|  | ***Acinetobacter* spp.** | 0/2 | 0/2 | 1/2 | 0/2 |
|  | **Other non-fermentative gram-negative bacteria** |  |  |  |  |
|  | *Achromobacter xylosoxidans* | 0/1 | 0/1 | 1/1 | 0/1 |
|  | **Other gram-negative bacteria** |  |  |  |  |
|  | *Capnocytophaga sputigena*** | 1/1 | 1/1 | 1/2 | NA |
|  | *Moraxella osloensis* | NA | 0/1 | NA | NA |
|  | **Anaerobic Gram-negative bacteria** |  |  |  |  |
|  | *Fusobacterium* spp. | NA | NA | NA | NA |
|  | **Total** | **14/19** | **13/19** | **5/20** | **0/17** |

*Displayed resistance was based on susceptibility testing; all were regarded as 3GC-R due to chromosomal AmpC.

**Ceftriaxone resistant; isolate not tested for ceftazidime, one isolate not tested for ciprofloxacin and co-trimoxazole

Supplementary table 3; Empirical antibiotic therapy

|  | MTC | CTC | Total |
| --- | --- | --- | --- |
| FN episodes | 348 | 325 | 673 |
| Duration of systemic antibiotic therapy in days, median (IQR) | 8 (6) | 4 (4) | 6 (6) |
| Choice of empirical therapy   - Ceftazidime - Meropenem - Other* | 14/348 (4.0)  329/348 (94.5)  5/348 (1.4) | 285/325 (87.7)  34/325 (10.5)  6/325 (1.8) | 299/673 (44.4)  363/673 (53.9)  11/673 (1.6) |

*Cefotaxime, Amikacine, Ceftolozane/tazobactam, Ceftriaxone, Imipenem/cilastatine

Supplementary table 4; Generalized Estimating Equations analysis of BSI, 3GC-R EP BSI and IEAT for the composite outcome all-cause mortality/ ICU-transfer within 30-days after FN onset.

|  | **ICU/mortality within 30 days** | **Total** | **OR** | **95% CI** | **p-value** |
| --- | --- | --- | --- | --- | --- |
|  | YES (% of Total) |  |  |  |  |
| **BSI** | 22 (16.3) | 135 | 1.0803 | -0.5405  0.6951 | 0.806 |
| **No BSI** | 75 (13.9) | 538 |  |  |  |
| **3GC-R EP BSI** | 2 (11.8) | 17 | 0.4396 | -2.9645  1.3205 | 0.452 |
| **No 3GC-R EP BSI** | 95 (14.5) | 656 |  |  |  |
| **IEAT** | 7 (25.9) | 27 | 1.9048 | -0.4550  1.7437 | 0.251 |
| **No IEAT** | 90 (13.9) | 646 |  |  |  |
| **Total** | 97 (14.4) | 673 |  |  |  |

BSI = blood-stream infection, 3GC-R EP = third-generation cephalosporin resistant Enterobacterales and *Pseudomonas aeruginosa,* IEAT = Inappropriate empirical antimicrobial therapy.
